# Effectiveness of implantable loop recorder and Holter electrocardiographic monitoring for the detection of arrhythmias in patients with peripartum cardiomyopathy

**DOI:** 10.1007/s00392-022-02101-3

**Published:** 2022-09-22

**Authors:** Julian Hoevelmann, Karen Sliwa, Olivia Briton, Mpiko Ntsekhe, Ashley Chin, Charle Viljoen

**Affiliations:** 1 Cape Heart Institute, Faculty of Health Sciences, University of Cape Town, 4th Floor Chris Barnard Building, Observatory, Private Bag X3, Cape Town, 7935 South Africa; 2 Klinik für Innere Medizin III, Kardiologie, Angiologie und Internistische Intensivmedizin, Universitätsklinikum des Saarlandes, Saarland University Hospital, Homburg (Saar), Deutschland; 3 Division of Cardiology, Faculty of Health Sciences, Groote Schuur Hospital, University of Cape Town, Cape Town, South Africa

**Keywords:** Peripartum cardiomyopathy, Arrhythmias, Extended electrocardiographic monitoring, Implantable loop recorder (ILR), 24 h-Holter monitoring, Sudden cardiac death

## Abstract

**Background:**

Patients with peripartum cardiomyopathy (PPCM) are at increased risk of sudden cardiac death (SCD). However, the exact underlying mechanisms of SCD in PPCM remain unknown. By means of extended electrocardiographic monitoring, we aimed to systematically characterize the burden of arrhythmias occurring in patients with newly diagnosed PPCM.

**Methods and results:**

Twenty-five consecutive women with PPCM were included in this single-centre, prospective clinical trial and randomised to receiving either 24 h-Holter ECG monitoring followed by implantable loop recorder implantation (ILR; REVEAL XT, Medtronic^®^) or 24 h-Holter ECG monitoring alone. ILR + 24 h-Holter monitoring had a higher yield of arrhythmic events compared to 24 h-Holter monitoring alone (40% vs 6.7%, *p* = 0.041). Non-sustained ventricular tachycardia (NSVT) occurred in four patients (16%, in three patients detected by 24 h-Holter, and multiple episodes detected by ILR in one patient). One patient deceased from third-degree AV block with an escape rhythm that failed. All arrhythmic events occurred in patients with a severely impaired LV systolic function.

**Conclusions:**

We found a high prevalence of potentially life-threatening arrhythmic events in patients with newly diagnosed PPCM. These included both brady- and tachyarrhythmias. Our results highlight the importance of extended electrocardiographic monitoring, especially in those with severely impaired LV systolic function. In this regard, ILR in addition to 24 h-Holter monitoring had a higher yield of VAs as compared to 24 h-Holter monitoring alone. In settings where WCDs are not readily available, ILR monitoring should be considered in patients with severely impaired LV systolic dysfunction, especially after uneventful 24 h-Holter monitoring.

**Trial registration:**

Pan African Clinical Trials Registry: PACTR202104866174807.

**Graphical abstract:**

Extended electrocardiographic monitoring for the detection of arrhythmias in PPCM. (CHB, complete heart block/third degree AV block; ECG, electrocardiogram; ILR, implantable loop recorder; NSVT, non-sustained ventricular tachycardia; PPCM, peripartum cardiomyopathy)

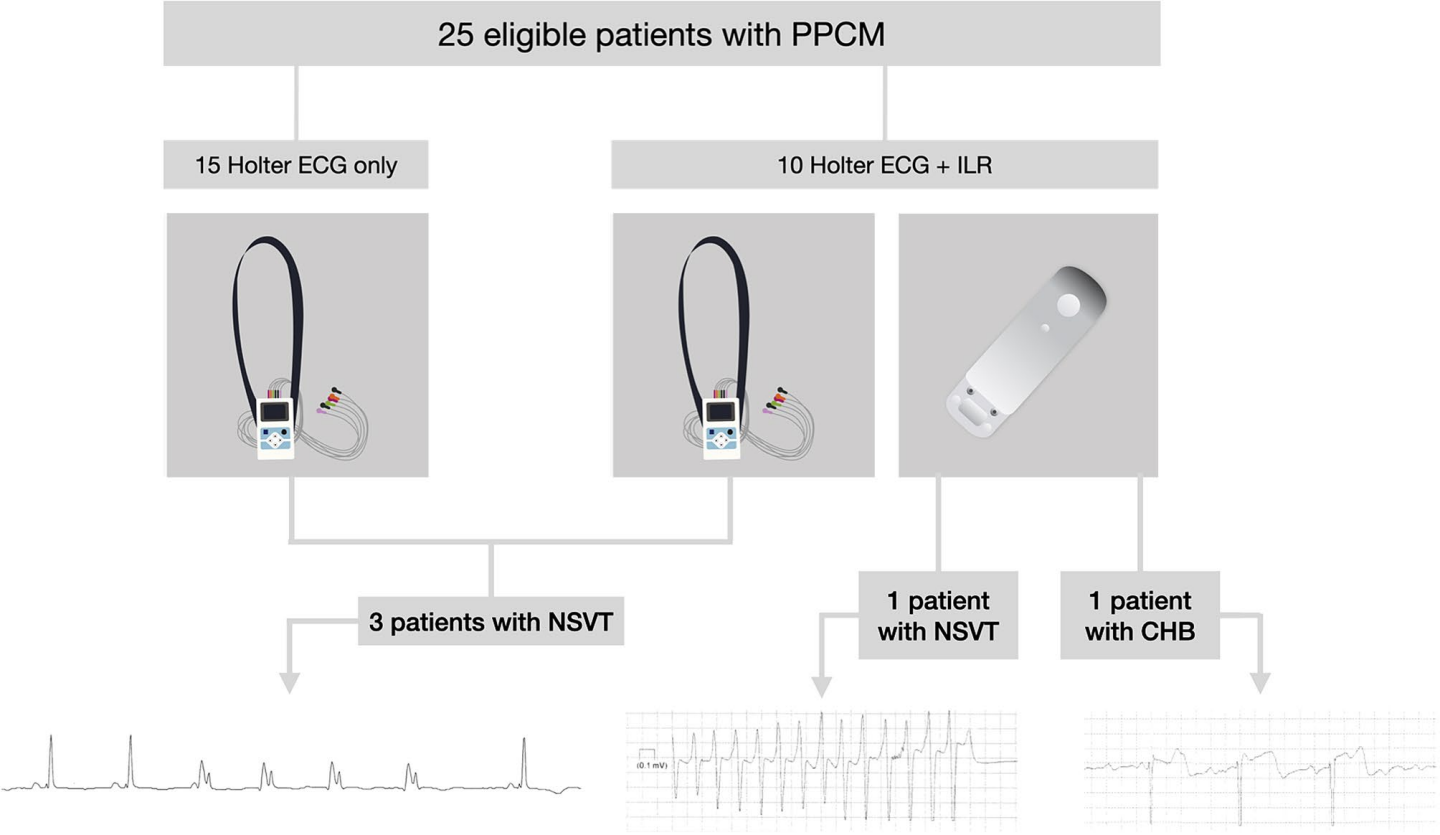

**Supplementary Information:**

The online version contains supplementary material available at 10.1007/s00392-022-02101-3.

## Introduction

Peripartum cardiomyopathy (PPCM) is a pregnancy-associated form of heart failure. The disease is defined as new-onset left ventricular (LV) systolic dysfunction (i.e., LV ejection fraction [LVEF] ≤ 45% at time of presentation, in the absence of pre-existing heart disease), which occurs towards the end of pregnancy or within five months postpartum [1]. The aetiology of PPCM is multifactorial. Recent studies suggest a ‘two-hit’ model, in which systemic angiogenic imbalance and host susceptibility (predisposition) are the key drivers in the pathogenesis of PPCM [2].

The European Observational Research Programme (EORP), which included 739 women with PPCM from 49 countries, reported a 6-month all-cause mortality of 6%, of which 30% were thought to be related to sudden cardiac death (SCD) [3]. Although a severely reduced LVEF is associated with an increased risk of life-threatening ventricular arrhythmias (VAs) in idiopathic dilated cardiomyopathy (DCM) [4], literature on the burden of arrhythmias in PPCM and their contribution to SCD remains sparse. In a retrospective analysis of 9841 hospital admissions for PPCM, Mallikethi-Reddy et al*.* reported a prevalence of 18.7% for arrhythmias during the time of hospitalisation. Ventricular tachycardia (VT) was the most common arrhythmia (4.2%), followed by atrial fibrillation (AF) (1.3%) and ventricular fibrillation (VF) (1%) [5]. However, in this cohort, the prevalence of arrhythmias outside the hospital setting is not known. In a study from Senegal, Diao et al*.* performed 24-h Holter ECG monitoring on 19 patients with PPCM. Sinus tachycardia, which is known to be associated with adverse outcome [6], was documented in almost 90% of their patients. Premature ventricular complexes (PVCs) occurred in seven patients, whereas one patient had four episodes of non-sustained ventricular tachycardia (NSVT) [7]. However, all 24 h-Holters were performed during the hospital stay and, therefore, only reflect the peri-diagnosis setting of PPCM.

In a study on the usefulness of the wearable cardioverter defibrillator (WCD) in PPCM, Duncker et al*.* found a high prevalence of life-threatening VAs in patients with severely reduced LVEF. Amongst three of the seven patients that received a WCD in this study, there were four VF episodes detected and successfully terminated by shock delivery [8]. These results were confirmed in a larger cohort, comprising 49 patients with newly diagnosed PPCM and severely reduced LVEF. The WCD detected VAs in six patients (12%) with a total of eight VAs (i.e., five episodes of VF, two sustained VTs and one NSVT) [9]. However, the above-mentioned studies only included PPCM patients who had an LVEF ≤ 35% at time of diagnosis.

There is currently limited knowledge regarding the screening and management of arrhythmias in women with PPCM. We, therefore, aimed to determine the burden of arrhythmias in PPCM using extended electrocardiographic monitoring beyond the time of diagnosis, not limited to patients with severely impaired LVEF (i.e., LVEF ≤ 35%). We also intended to compare the utility of implantable loop recorder (ILR) plus 24 h-Holter ECG monitoring versus 24 h-Holter ECG monitoring alone for the detection of arrhythmias in PPCM.

## Methods

### Study design

**Fig. 1:**
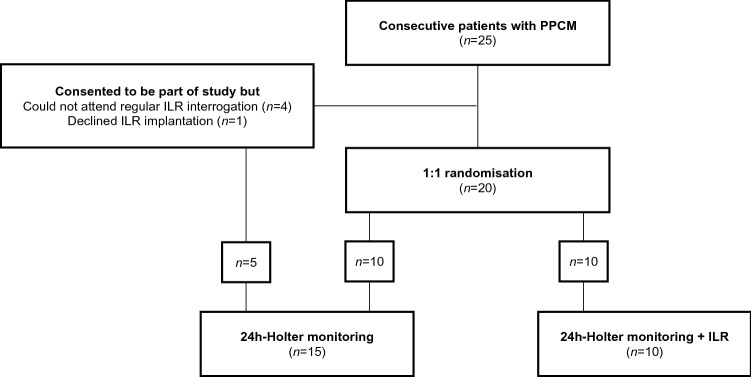
Study flow. *ILR* implantable loop recorder

From 2017 to 2021, we enrolled 25 consecutive, consenting patients with PPCM that presented to our dedicated heart failure clinic at Groote Schuur Hospital (GSH) and the University of Cape Town (UCT). In this single-centre, prospective clinical trial, patients were randomized to receive either 24 h-Holter monitoring plus an implantable loop recorder (ILR) (*n* = 10) or 24 h-Holter monitoring alone (*n* = 10). Whenever patients refused the ILR implantation (*n* = 1), or could not return to the hospital for regular ILR interrogations (e.g., they lived too far from the hospital, *n* = 4), they would still receive 24 h-Holter monitoring as part of routine care, and be included in the study (in addition to those that were randomized to 24 h-Holter monitoring alone). The study flow is outlined in Fig. 1. Apart from those who were lost to follow-up (*n* = 4), or died (n = 1), all patients returned for at least 2 years follow-up after recruitment to the study.

Ethical approval was obtained from the Human Research Ethics Committee (HREC) at UCT (HREC Ref. 178/2014) and the trial was registered with the Pan African Clinical Trials Registry (PACTR202104866174807). The study was performed in accordance with the Declaration of Helsinki and all patients provided written informed consent prior to study inclusion.

### Management and follow-up

Baseline data, including age, medical and obstetric history, clinical presentation (including New York Heart Association [NYHA] functional class [FC]), prescribed medication, 12-lead ECG, and transthoracic echocardiogram were recorded at the first visit.

All patients received 24 h-Holter monitoring with a continuous 2-channel ECG (SEER Light recorder, General Electric). Heart rate (including minimum, average, maximum rates), proportion of time in bradycardia (i.e., ≤ 60 bpm) or tachycardia (i.e., ≥ 100 bpm), burden of premature atrial and/or ventricular complexes (PACs, PVCs), as well as occurrence of supraventricular and/or VAs over the 24 h monitoring period were recorded. Non-sustained VT was defined as ≥ 3 ventricular beats at ≥ 100 bpm beats per minute detected on 24 h-Holter monitoring.

As per randomisation schedule, ten patients received an ILR (REVEAL XT, Medtronic®, Midrand, South Africa). The device was implanted under local anaesthetic and inserted subcutaneously in the left parasternal area of the chest, as guided by pre-implantation testing. The ILR was interrogated at the regular follow-up visits or when patients were symptomatic. The ILR would detect episodes of tachycardia and define these as either atrial tachycardia, atrial fibrillation or VT. As per out of box settings of the device, the ILR diagnostic algorithm detected VT when ≥ 16 ventricular beats had a VT interval of ≤ 340 ms. Episodes of bradycardia were detected when the bradycardia interval was ≥ 2000 ms for ≥ 4 s and asystole when no QRS complexes were detected for ≥ 3 s. Patients were asked to trigger the ILR remote button, when symptomatic of palpitations, dizziness or pre-syncope. All 24 h-Holter recordings and ILR readings (with a documented ECG strip for adjudication) were reviewed by an electrophysiologist and cardiologist (AC) and two fellows in Cardiology (JH, CV). Patients were followed-up for at least 12 months and up to 3 years. Poor outcome was determined as a composite of all-cause death, heart transplantation, readmission to hospital, or failure to show an LV improvement of > 10 units at follow-up.

### Statistical analysis

Statistical analyses were performed with Stata (Version 17, StataCorp, College Station, TX, USA). Descriptive statistics were used to summarise data. Distribution of data was determined by Shapiro–Wilk test. Continuous variables were summarised as means with standard deviations (SD) for parametric data or median with interquartile range (IQR) for non-parametric data. Categorical variables were expressed as frequencies and percentages. Where appropriate, we used a Kruskal–Wallis or Wilcoxon rank-sum test (for continuous variables) and chi-squared or Fisher’s exact test (for categorical variables), to compare patients assigned to ILR + 24 h-Holter monitoring compared to 24 h-Holter monitoring alone as well as patients with and without arrhythmic events. Correlations between 12-lead ECG and 24 h-Holter monitoring were assessed using Spearman’s rank test. A *p* value of < 0.05 was considered to indicate statistical significance.

## Results

As depicted in Table 1, this cohort of 25 women with PPCM had a mean age of 30.4 ± 6.6 years with a median parity of 1 (interquartile range [IQR] 1–4). Most of the patients presented with severe acute heart failure, with 88% of patients having NYHA FC III or IV. The mean LVEF at presentation was 28.6 ± 10.8% and 84% of the cohort presented with an LVEF ≤ 35% at time of diagnosis. The median LVEDD was 59 mm (IQR 55–65) and 76% had LV dilatation (i.e., LVEDD ≥ 53 mm). Treatment at discharge from hospital consisted of angiotensin-converting enzyme inhibitors (ACE-I)/angiotensin receptor blockers (ARBs) (80%), beta-blockers (80%), mineralocorticoid-receptor antagonists (MRA) (52%), diuretics (88%) and bromocriptine (52%).

**Table 1:** Baseline characteristics of study population, as categorised by receiving ILR or not, and by having had detected arrhythmia or not

	Total	ILR and Holter	Holter only	*p* value	Arrhythmia detected	No arrhythmia detected	*p* value
	*N* = 25	*N* = 10	*N* = 15		*N* = 5	*N* = 20	
Age (years)	30.4 ± 6.6	30.3 ± 4.9	30.4 ± 7.6	0.99	32.1 ± 5.4	29.9 ± 6.9	0.53
Parity	1.0 (1.0–4.0)	1.5 (1.0–4.0)	1.0 (1.0–4.0)	0.79	3.0 (1.0–4.0)	1.0 (1.0–4.0)	0.69
NYHA functional class at presentation				1.00			0.66
I	1 (4.0)	0 (0.0)	1 (6.7)		0 (0.0)	1 (5.0)	
II	2 (8.0)	1 (10.0)	1 (6.7)		1 (20.0)	1 (5.0)	
III	10 (40.0)	4 (40.0)	6 (40.0)		2 (40.0)	8 (40.0)	
IV	12 (48.0)	5 (50.0)	7 (46.7)		2 (40.0)	10 (50.0)	
BMI (kg/m^2^)	27.8 ± 7.5	26.8 ± 8.2	28.6 ± 7.3	0.60	27.9 ± 6.2	27.8 ± 7.9	0.99
SBP at presentation (mmHg)	119.6 ± 21.1	109.3 ± 22.5	126.4 ± 17.7	0.044	108.6 ± 7.6	122.3 ± 22.6	0.20
DBP at presentation (mmHg)	77.4 ± 14.4	72.7 ± 11.8	80.6 ± 15.5	0.19	70.0 ± 6.4	79.3 ± 15.4	0.20
QRS rate at presentation (bpm)	100.8 ± 20.1	96.6 ± 24.7	103.5 ± 16.7	0.41	91.6 ± 25.8	103.1 ± 18.5	0.26
Rhythm at presentation				0.51			0.29
Sinus rhythm	9 (36.0)	4 (40.0)	5 (33.3)		1 (20.0)	8 (40.0)	
Bradycardia	1 (4.0)	1 (10.0)	0 (0.0)		1 (20.0)	0 (0.0)	
Tachycardia	15 (60.0)	5 (50.0)	10 (66.7)		3 (60.0)	12 (60.0)	
T wave inversion	18 (72.0)	5 (50.0)	13 (86.7)	0.045	3 (60.0)	15 (75.0)	0.50
QTc by Bazett (ms)	469.6 ± 35.5	476.2 ± 33.2	465.2 ± 37.4	0.46	473.7 ± 30.4	468.6 ± 37.3	0.78
Prolonged QTc	14 (56.0)	7 (70.0)	7 (46.7)	0.25	4 (80.0)	10 (50.0)	0.23
LVEF at presentation (%)	28.6 ± 10.8	26.5 ± 12.3	30.0 ± 10.0	0.44	21.2 ± 5.4	30.5 ± 11.2	0.088
LVEF ≤ 35% at presentation	21 (84.0)	9 (90.0)	12 (80.0)	0.50	5 (100.0)	16 (80.0)	0.28
LVEDD at presentation (mm)	59.0 (55.0–65.0)	64.5 (59.0–67.5)	55.0 (48.0–61.0)	0.026	66.0 (60.0–67.0)	57.5 (50.0–62.0)	0.052
LVESD at presentation (mm)	53.0 (47.0–56.0)	56.5 (53.0–58.5)	48.0 (44.0–54.0)	0.017	58.0 (53.0–60.0)	49.0 (44.5–55.5)	0.025
Average heart rate in 24 h-Holter	95.0 (85.0–99.0)	90.0 (85.0–95.0)	96.0 (89.0–103.0)	0.071	95.0 (85.0–99.0)	94.5 (86.5–98.0)	0.84
Number of PVCs on 24 h-Holter	0.0 (0.0–0.0)	0.0 (0.0–0.0)	0.0 (0.0–0.0)	0.81	0.0 (0.0–0.0)	0.0 (0.0–0.0)	1.00
Number of PACs on 24 h-Holter	0.0 (0.0–0.0)	0.0 (0.0–0.0)	0.0 (0.0–0.0)	0.41	0.0 (0.0–0.0)	0.0 (0.0–0.0)	0.62
Time in tachycardia	31.0 (13.0–46.0)	24.0 (12.0–31.0)	38.0 (13.0–57.0)	0.19	39.0 (21.0–50.0)	30.5 (12.5–42.5)	0.59
Non-sustained VT on 24 h-Holter	3 (12.0)	2 (20.0)	1 (6.7)	0.31	3 (60.0)	0 (0.0)	< 0.001
Time in bradycardia	0.0 (0.0–0.0)	0.0 (0.0–0.0)	0.0 (0.0–0.0)	0.54	0.0 (0.0–0.0)	0.0 (0.0–0.0)	0.67
Furosemide	22 (88.0)	9 (90.0)	13 (86.7)	0.80	5 (100.0)	17 (85.0)	0.36
Spironolactone	13 (52.0)	6 (60.0)	7 (46.7)	0.51	3 (60.0)	10 (50.0)	0.69
ACE-i or ARB	20 (80.0)	9 (90.0)	11 (73.3)	0.31	4 (80.0)	16 (80.0)	1.00
Beta-blocker	20 (80.0)	9 (90.0)	11 (73.3)	0.31	3 (60.0)	17 (85.0)	0.21
Digoxin	1 (4.0)	0 (0.0)	1 (6.7)	0.40	0 (0.0)	1 (5.0)	0.61
Bromocriptine	13 (52.0)	7 (70.0)	6 (40.0)	0.14	3 (60.0)	10 (50.0)	0.69
Warfarin	4 (16.0)	2 (20.0)	2 (13.3)	0.66	2 (40.0)	2 (10.0)	0.10

Data are presented as mean ± SD or median (IQR) for continuous measures, and *n* (%) for categorical measures

*ACE-i* angiotensin-converting enzyme inhibitors, *ARB* angiotensin receptor blockers, *BMI* body mass index, *DBP* diastolic blood pressure, *LVEDD* left ventricular end-diastolic diameter, *LVEF* left ventricular ejection fraction, *LVESD* left ventricular end-systolic diameter, *NSVT* non-sustained ventricular tachycardia, *NYHA FC* New York Heart Association functional class, *PVCs* premature ventricular complexes, *QTc* corrected QT interval, *SBP* systolic blood pressure

Extended electrocardiographic monitoring documented major arrhythmic events in five patients. 24 h-Holter ECG monitoring documented three episodes of NSVT in three different patients. The REVEAL ILR detected multiple episodes of NSVT in the same patient and one third degree atrioventricular (AV) block with ventricular escape, which eventually failed and deteriorated to an agonal rhythm. Patients randomised to the ILR + 24 h-Holter monitoring arm had a more pronounced LV dilatation (LVEDD 64.5 mm [59.0–67.5] vs. 55.0 [48.0–61.0], *p* = 0.026), however, the LVEF was not different between the two groups. Moreover, ILR + 24 h-Holter monitoring had a higher yield of arrhythmic events compared to 24 h-Holter monitoring alone (40% vs 6.7%, *p* = 0.041). There were no differences in LVEF or LV dimensions at follow-up between the two groups (Table 2). No AT or AF was documented in any patient in this cohort. Patients who had arrhythmic events presented with a higher LVESD 58.0 mm [53.0–60.0] vs 49.0 mm [44.5–55.5]; *p* = 0.025) and tended to have higher initial LVEDD (66.0 mm [60.0–67.0] vs 57.5 mm [50.0–62.0]; *p* = 0.052) as well as lower LVEF (21.2 ± 5.4 vs 30.5 ± 11.2%; *p* = 0.088) at baseline. The other baseline characteristics of patients with or without arrhythmic events were similar (Table 1).

**Table 2:** Clinical, ECG and echocardiographic findings at follow-up, as categorised by receiving ILR or not, and by having had detected arrhythmia or not

	Total	ILR and Holter	Holter only	*p* value	Arrhythmia detected	No arrhythmia detected	*p* value
	*N* = 25	*N* = 10	*N* = 15		*N* = 5	*N* = 20	
NYHA functional class at follow-up				0.38			0.84
I	6 (54.5)	3 (60.0)	3 (50.0)		1 (50.0)	5 (55.6)	
II	4 (36.4)	1 (20.0)	3 (50.0)		1 (50.0)	3 (33.3)	
III	1 (9.1)	1 (20.0)	0 (0.0)		0 (0.0)	1 (11.1)	
Heart rate at follow-up (bpm)	70.5 (66.0–80.0)	70.0 (64.0–71.0)	77.0 (66.0–84.0)	0.25	67.0 (64.0–70.0)	74.0 (66.0–82.0)	0.29
SBP at follow-up (mmHg)	119.0 (110.0–130.0)	120.0 (118.0–128.0)	115.0 (100.0–130.0)	0.46	124.0 (120.0–128.0)	116.5 (105.0–131.0)	0.60
DBP at follow-up (mmHg)	65.5 (60.0–80.0)	70.0 (60.0–80.0)	64.0 (60.0–67.0)	0.59	70.0 (60.0–80.0)	65.5 (60.0–77.5)	0.89
QRS rate at follow-up (ms)	74.9 ± 12.4	74.4 ± 15.6	75.4 ± 10.1	0.91	82.0 ± 26.9	73.1 ± 8.8	0.40
Rhythm at follow-up				0.44			0.022
Sinus rhythm	8 (80.0)	3 (60.0)	5 (100.0)		0 (0.0)	8 (100.0)	
Tachycardia	1 (10.0)	1 (20.0)	0 (0.0)		1 (50.0)	0 (0.0)	
Sinus arrhythmia	1 (10.0)	1 (20.0)	0 (0.0)		1 (50.0)	0 (0.0)	
T wave inversion at follow-up	6 (60.0)	3 (60.0)	3 (60.0)	1.00	0 (0.0)	6 (75.0)	0.053
QTc at follow-up by Bazett (ms)	449.7 ± 25.7	432.1 ± 12.8	473.1 ± 17.2	0.015	431.3 ± 4.5	457.0 ± 27.4	0.27
Prolonged QTc at follow-up	2 (28.6)	0 (0.0)	2 (66.7)	0.053	0 (0.0)	2 (40.0)	0.29
LVEF at follow-up	45.8 ± 17.2	51.4 ± 17.9	41.9 ± 17.0	0.37	55.3 ± 23.9	42.7 ± 14.9	0.29
LV improvement of > 10 units in percentage	21 (84.0)	9 (90.0)	12 (80.0)	0.50	5 (100.0)	16 (80.0)	0.28
LVEDD at follow-up (mm)	55.0 (48.5–60.0)	58.0 (52.0–62.0)	51.0 (48.0–58.0)	0.46	52.0 (44.0–65.0)	58.0 (49.0–58.0)	0.93
LVESD at follow-up (mm)	39.5 (34.0–50.0)	39.5 (30.0–45.0)	43.0 (34.0–51.0)	0.52	30.0 (22.0–38.0)	45.0 (35.0–50.5)	0.19
Arrhythmic event detected	5 (20.0)	4 (40.0)	1 (6.7)	0.041	5 (100.0)	0 (0.0)	< 0.001
Poor outcome	8 (32.0)	4 (40.0)	4 (26.7)	0.48	4 (80.0)	4 (20.0)	0.010
Death	1 (4.0)	1 (10.0)	0 (0.0)	0.21	1 (20.0)	0 (0.0)	0.041

Data are presented as mean ± SD or median (IQR) for continuous measures, and *n* (%) for categorical measures

*DBP* diastolic blood pressure, *LVEDD* left ventricular end-diastolic diameter, *LVEF* left ventricular ejection fraction, *LVESD* left ventricular end-systolic diameter, *NYHA FC* New York Heart Association functional class, *QTcB* corrected QT interval by Bazett’s formula, *SBP* systolic blood pressure

### 24-Holter monitoring

**Fig. 2:**
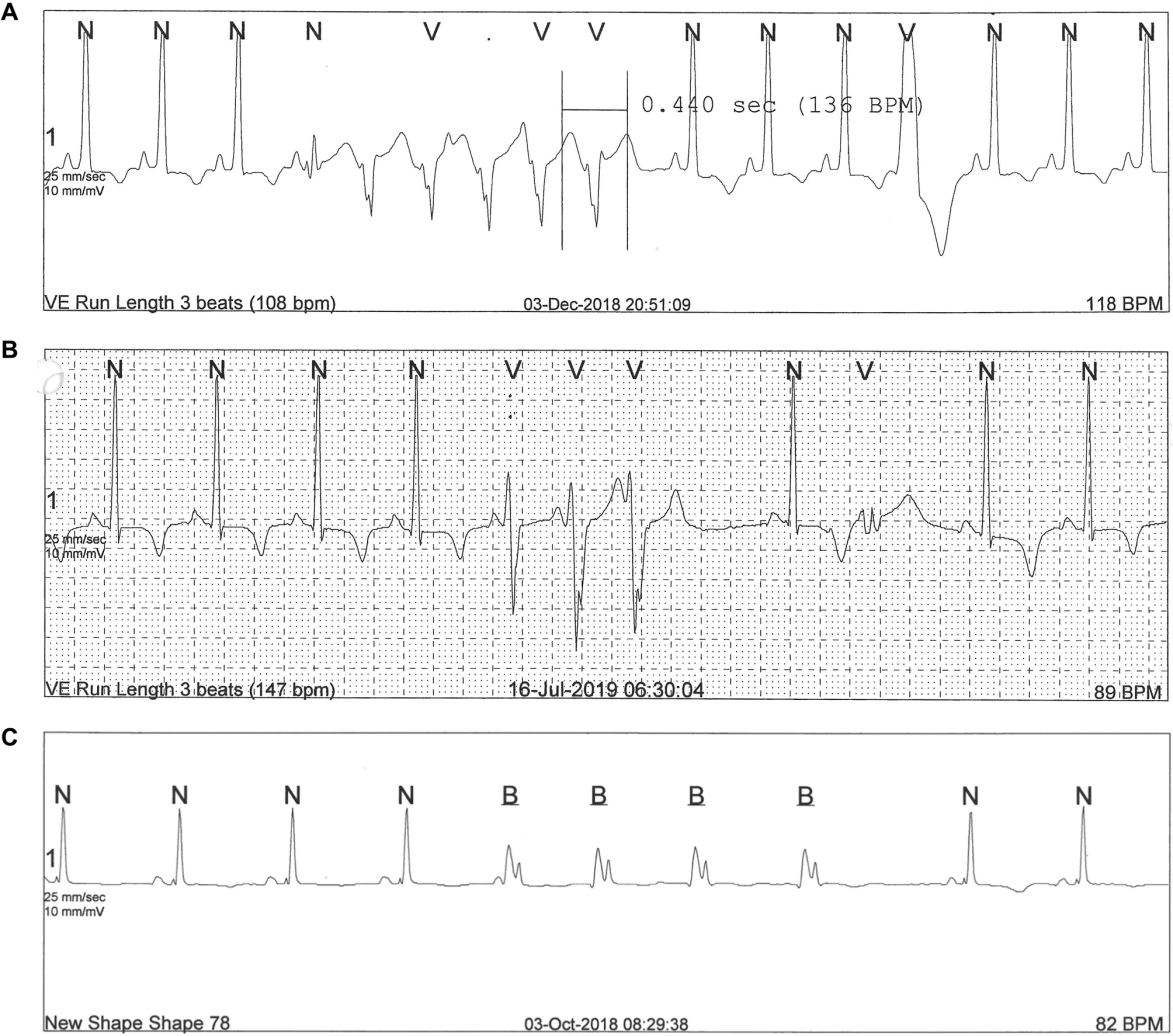

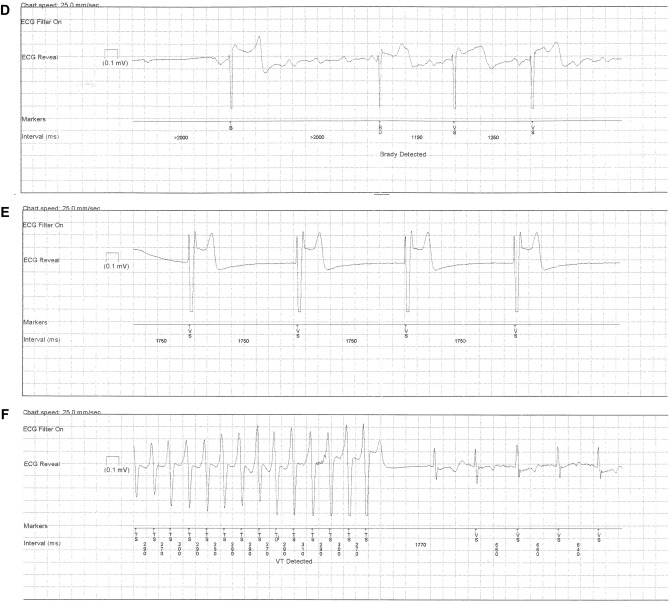
24-h Holter ECG monitoring of three patients with PPCM (**A**, **B**, **C**) showing episodes of non-sustained ventricular tachycardia. ILR interrogation revealed a third-degree AV block (**D**), which deteriorated to asystole with a brief episode of an agonal rhythm (**E**) in one patient, and multiple episodes of non-sustained, polymorphic ventricular tachycardia (**F**) in another patient. Both patients had an LVEF < 35% at the time of diagnosis

The median heart rate on 24-Holter ECG monitoring was 95 bpm (IQR 85–99) and time in tachycardia was 31% (IQR 13–46). There was a weak correlation between heart rate recorded by the 12-lead ECG and on 24 h-Holter ECG (*r* = 0.3529, *p* = 0.084)*.* For episodes where the heart rate exceeded 100 bpm, sinus tachycardia was the predominant condition seen. Mean PVC burden was low (72 ventricular beats/24 h [IQR 17–227]). Non-sustained VT was detected in three patients (12%). All three women, in whom NSVT was detected by 24 h-Holter ECG monitoring, presented with a severely impaired LVEF (12, 23 and 25%, respectively) (Fig. 2, Table 3). No episodes of AF/flutter, other supraventricular tachycardias or AV block were detected by the 24 h-Holter monitoring.

**Table 3:** Characteristics and follow-up of patients with documented arrhythmic events

	Patient #7	Patient #13	Patient #15	Patient #20	Patient #24
Age (years)	32	35	38	27	25
Time of presentation	Prepartum	Postpartum	Postpartum	Postpartum	Postpartum
Parity	4	1	4	3	1
NYHA FC at diagnosis	IV	III	III	II	IV
QTc interval by Bazett (ms)	424	478	469	503	494
LVEDD (mm)	66	59	60	67	69
LVEF at diagnosis (%)	21	12	25	23	25
LVEF at latest follow-up (%)	29	62%*	Not available	67	Deceased
∆LVEF (%)	8	50*	Not available	44	Deceased
Arrhythmia detected on 24 h-Holter or ILR	ILR	24 h-Holter	24 h-Holter	24 h-Holter	ILR
Arrhythmia type	NSVT	NSVT	NSVT	NSVT	Third degree AV block with subsequent asystole
Onset of first documented arrhythmia (after diagnosis)	2 weeks	1 week	1 week	1 week	14 weeks
Management	Primary prophylactic ICD implanted	Heart transplantation for intractable heart failure	Heart failure therapy	Heart failure therapy	Out-of hospital cardiac arrest

*AV* atrioventricular, *ICD* implantable cardioverter defibrillator, *ILR* implantable loop recorder, *LVEDD* left ventricular end-diastolic diameter, *LVEF* left ventricular ejection fraction, *NSVT* non-sustained ventricular tachycardia, *NYHA FC* New York Heart Association functional class

*Echocardiography at follow was of the transplanted heart

### Implantable cardiac rhythm monitors

**Fig. 3:**
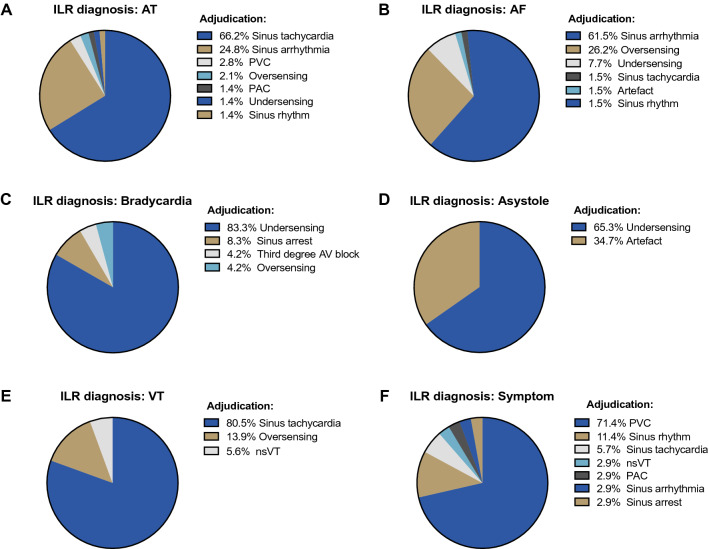
Overview of adjudication of automated ILR diagnoses. *AF* atrial fibrillation, *AT* atrial tachycardia, *AV* atrio-ventricular, *ILR* implantable loop recorder, *NSVT* non-sustained ventricular tachycardia, *PAC* premature atrial contraction, *PVC* premature ventricular contraction, *VT* ventricular tachycardia

The median duration of follow-up for the ten patients who received an ILR was 385 days (IQR 126–756). Overall, the ILR devices automatically detected 346 asymptomatic arrhythmic episodes with stored ECG documentation. Before adjudication, the ILR device classified 65 episodes as AF (17.1%), 145 episodes as AT (38.1%), 72 episodes as asystole (18.9%), 24 episodes as bradycardia (6.3%), 4 episodes as fast VT (FVT), and 36 episodes as VT (9.5%). As seen in Fig. 3A, B, the predominant adjudicated diagnosis for AT was sinus tachycardia (66.2%) and sinus arrhythmia for the ILR diagnosis of AF (61.5%). Importantly, there were no true episodes of AF or AT in any patient. All episodes classified as asystole by the device were adjudicated as being either ventricular undersensing or artefact (Fig. 3D). Of all episodes classified as VT by the ILR, only two episodes (5.6%) were adjudicated as being true NSVT, whereas the majority (80.5%) were adjudicated as sinus tachycardia (Fig. 3E). There were 35 episodes of patient documented symptoms. The most common adjudication for patient symptoms were PVCs (71.4%). One patient symptom represented a symptomatic NSVT episode (Fig. 3F). Examples of adjudicated ILR rhythm strips can be seen in Supplementary Fig. 1.

### Outcomes

Our study shows that arrhythmic events were associated with adverse outcome (Table 2). Indeed, in 80% of patients with an arrhythmia had poor outcome vs 20% in those without (*p* = 0.010).

Five participants had significant findings on either 24 h-Holter monitoring or ILR (Table 3). One patient (#24) suffered an out-of-hospital sudden cardiac death 1 week after ILR-implantation. Her ILR reading initially showed third degree AV block. The associated ventricular escape rhythm deteriorated to asystole with a brief period of an agonal rhythm (Fig. 2, recording D, E). Unfortunately, she died before reaching the emergency department. At the time of PPCM diagnosis, her echocardiogram showed a dilated LV (LVEDD 69 mm) with severely impaired LV systolic function (25%) and LV thrombus. Her initial 24 h-Holter ECG documented no ventricular brady- or tachyarrhythmias. Medication at discharge included Enalapril, Carvedilol, Spironolactone, Bromocriptine and Warfarin.

A second patient (#7) had multiple, symptomatic episodes of non-sustained, polymorphic VT detected by ILR (Fig. 2, recording F). At her initial presentation, she required mechanical ventilation due to severe pulmonary oedema and had a dilated left ventricle (LVEDD 59 mm) with severely impaired LVEF (21%). Her 24 h-Holter ECG monitoring, however, showed no evidence of VAs. The first documented episode of NSVT was detected about four months after ILR implantation. Due to persistent LV systolic dysfunction, despite more than three months of optimal medical therapy (OMT) and multiple episodes of NSVT recorded by ILR, the patient received an implantable cardioverter-defibrillator (ICD) for secondary prevention. At her latest follow-up the patient still showed no recovery of LV systolic function (Table 3). She has received no ICD shocks since device implantation.

A third patient (#13) presented with acute severe heart failure with an LVEF of 12%. Her 24 h-Holter ECG documented an episode of NSVT (Fig. 2, recording C). The patient later underwent successful heart transplantation due to intractable heart failure with recurrent hospital admissions.

A fourth participant showed recovery of her LV systolic function within 6 months (#20) and one patient was lost to follow-up (#15).

Patients with NSVT presented with a lower LVEF at baseline 20.2 ± 5.7% vs 30.2 ± 10.9%. p = 0.093). Indeed, all patients with NSVT detected by 24 h-Holter ECG monitoring or ILR had an LVEF ≤ 35% at presentation.

## Discussion

In this study on extended electrocardiographic monitoring in PPCM, we showed a high prevalence of arrhythmic events. In this regard, ILR in addition to 24 h-Holter monitoring had a significantly higher arrhythmic yield than 24 h-Holter monitoring alone. All major brady- and tachyarrhythmias occurred in the early phase of the disease (i.e., within the first 4 months after diagnosis). Neither 24 h-Holter or ILR monitoring detected any episodes of AF.

The most commonly detected VA in this cohort was NSVT, which occurred in four patients (16%), all of which had a severely impaired LVEF. Our reported prevalence of VAs is comparable to studies on the use of the WCD in patients with newly diagnosed PPCM, which also showed a high burden of VAs (12%) [9]. Previous studies have shown that a prolonged corrected QT (QTc) interval by Bazett’s formula (QTcB) is associated with adverse outcome in PPCM [6]. Indeed, it is well established that a prolonged QTc predisposes to VAs and is associated with SCD [10]. Three out of four patients with NSVT had a prolonged QTcB at presentation. However, our study was underpowered to show a conclusive relationship between QT prolongation and occurrence of VAs.

In a recent systematic review and meta-analysis, Sammani et al*.* [11] evaluated the burden of VAs in patients with non-ischaemic DCM. They reported an annual event rate of 4.5% for sustained VAs in DCM. In their study, younger age, hypertension, prior (non-)sustained VA, decreased LVEF, LV dilatation, late gadolinium enhancement (LGE) and genetic mutations (Phospholamban *(PLN),* Lamin A/C *(LMNA)*, and Filamin-C *(FLNC)*) were significant predictors of arrhythmic events. [11]

One patient with severe heart failure developed third degree AV block 1 week after PPCM diagnosis. The ventricular escape rhythm eventually failed, and the patient demised from asystole before she could reach the hospital. Complete heart block (CHB) at the time of PPCM diagnosis has previously only been described in case reports [12, 13]. In both case reports, an underlying myocarditis with focal inflammation was suggested as a possible mechanism of the CHB. However, this was not confirmed by cardiac magnetic resonance (CMR) in either of the cases [12, 13]. In a cohort of patients with advanced heart failure, severe bradycardia or electromechanical dissociation (EMD) was described as the rhythm at the time of arrest in 62% of patients [14]. Severe bradyarrhythmia or EMD, in particular, precedes cardiac arrest in patients with idiopathic DCM, and is believed to account for about one-third SCD in these patients. [15, 16]

24 h-Holter ECG monitoring is an inexpensive and non-invasive diagnostic modality, but is confined to monitoring during the usual 24–48 h weartime [17]. However, newer devices (i.e., adhesive patches attached to a small device recorder) allow for continuous rhythm monitoring over several days [18]. The benefit of ILRs is that they allow for much longer periods of rhythm monitoring (i.e., up to 3 years). The device records patient- or event-activated (auto-triggered) ECG tracings. ILRs are usually indicated, if the suspected arrhythmia occurs infrequently. Disadvantages are the invasive insertion with the risk of postoperative wound infection, and higher costs compared to Holter ECG monitoring or patches [19]. Furthermore, ILRs do not typically record short runs of NSVT. The REVEAL XT^©^ device that we used in this study, only detected NSVT when more than 16 consecutive beats of NSVT was detected by the device. The prevalence of NSVT might, therefore, be higher than what we report.

In this study, we could show that ILR in addition to Holter monitoring had a higher yield of arrhythmic events than 24 h-Holter monitoring alone. We also found that patients with severely impaired LV systolic function were at highest risk of arrhythmias, particularly shortly after index diagnosis. We, therefore, recommend that ILR should be considered in the early post-partum period in patients with severe heart failure and/or severe LV dysfunction, especially in patients with symptoms suggestive of an arrhythmia, and particularly when the 24 h-Holter did not detect any arrhythmia. However, larger prospective studies are required to support our recommendations.

Our study also highlights the importance of event adjudication of the REVEAL XT^©^ device. In our study, the minority of detected arrhythmias were true arrhythmic events. As our study population consisted of pregnant and postpartum women, some false events may be related to enlarged breasts leading to artefacts. However, regular device interrogations and expert adjudications of all stored events are crucial to detect arrhythmic events. Newer generation ILR devices can be connected telemonitoring platforms and allow for a more timely response to a serious arrhythmic event and give the opportunity for pre-emptive interventions [19].

Previous prospective studies on the prevalence of major arrhythmic events in PPCM investigated the use of a WCD [8, 9]. Similar to our study, Duncker et al*.* demonstrated that arrhythmic events occurred in the early phase after PPCM diagnosis (40–165 days). In their study, the WCD detected VAs in 12% of patients and all episodes of VF could be successfully terminated by a device shock [9]. These data highlight that WCD has important therapeutic benefit in terminating ventricular tachyarrhythmias in patients with severely reduced LVEF. Indeed, WCD can, therefore, be used as “bridging” therapy, allowing for medical therapy to be initiated and protecting against SCD from VAs. This strategy would prevent unnecessary implantation of ICDs in young women with a disease with potential for LV recovery [20]. However, due to the high cost of the device, patient selection and risk stratification is of great importance [21].

**Fig. 4:**
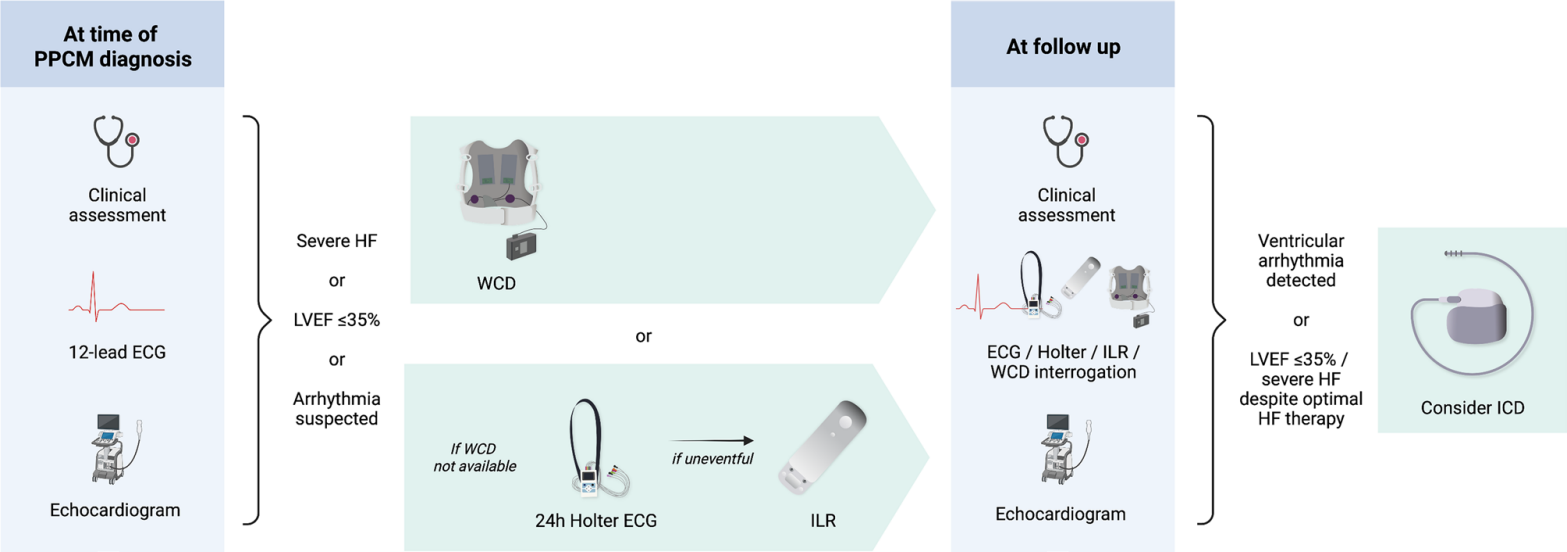
Proposed algorithm for the diagnosis and management of arrhythmias in PPCM. *ECG* electrocardiogram, *HF* heart failure, *ICD* implantable cardioverter defibrillator, *ILR* implantable loop recorder, *LVEF* left ventricular ejection fraction, *WCD* wearable cardioverter defibrillator

It should be highlighted that WCDs are not available in large parts of the world. As illustrated in the proposed algorithm for the detection and management of arrhythmias (Fig. 4), we recommend extended electrocardiographic monitoring in PPCM if WCDs are not available. This would include Holter monitoring on all patients with severely impaired LV systolic function in the acute phase of the disease, and ILRs when 24 h-Holter monitoring was inconclusive, or if symptoms suggestive of arrhythmias occur infrequently.

### Limitations

We acknowledge that the patient cohort included in this study is small, and do not allow for definitive recommendations. However, PPCM is a relatively rare disease. The sample size might have affected the estimate of the true prevalence of arrhythmias in patients with newly diagnosed PPCM. As only ten patients received an ILR (which allows for continuous rhythm monitoring), we cannot comment on subsequent arrhythmias in patients who only received 24 h-Holter ECG monitoring.

Our study is also not adequately powered to answer the question about which patients are most likely to benefit from ILR, but patients with severely reduced LV function or severe symptoms of heart failure appeared to be at higher risk for ventricular arrhythmias. Therefore, as recommended by the latest ESC position statement on PPCM [2], WCDs should be considered for patients with a LVEF ≤ 35% in the early stage of disease, i.e., when arrhythmias are more prevalent. However, these devices are not readily available in large parts of the world. In settings where WCDs are unavailable for patients with a LVEF ≤ 35%, extended monitoring with an ILR may, therefore, be useful for the detection of atrial and ventricular arrhythmias. If ventricular arrhythmias are detected, an ICD should be considered for secondary prevention.

Our sample size is also too small to answer the question whether patients with short runs of NSVT have a higher risk of SCD. However, it may be a surrogate marker of a more severely diseased ventricle. The detection of NSVT is important, because it has previously been reported to be a marker of increased risk of SCD in patients with non-ischaemic cardiomyopathy [22]. Late gadolinium enhancement (LGE) on cardiovascular magnetic resonance (CMR) has been established as a strong predictor of VAs and SCD in a wide spectrum of non-ischaemic cardiomyopathies [23]. We acknowledge the lack of CMR scans in this cohort, which could have been important to answer the question if PPCM patients with arrhythmic events show signs of structural abnormalities such as LGE on CMR.

In this study, we used the out of the box settings for the detection of VAs by ILR, as most physicians would do in clinical practice. It is possible this underestimated the frequency of shorter NSVT episodes. However, considering only NSVT of more than 16 consecutive beats on Holter, would have decreased the detection rate by Holter monitoring.

A further limitation to this study is that Holter monitoring was only performed at the time of index diagnosis, and not at follow-up. This might have underestimated the true prevalence of arrhythmias in this cohort.

We encourage validation of our findings in a larger, multi-centred cohort. However, to the best of our knowledge, this is the largest prospective study on extended electrocardiographic monitoring on consecutive women with PPCM regardless of their initial LVEF.

## Conclusions

In this study on extended electrocardiographic monitoring in patients with PPCM, we observed a high prevalence of arrhythmic events. Importantly, these arrhythmias included both tachy- and bradyarrhythmias. Arrhythmias occurred predominantly early after diagnosis and in patients with a severe acute heart failure. In this regard, ILR monitoring in addition to 24 h-Holter monitoring had a higher yield of VAs as compared to 24 h-Holter monitoring alone. We could show that extended electrocardiographic monitoring had a direct influence on clinical decision making and outcome. In settings where WCDs are not readily available, ILR monitoring should be considered in patients with severely impaired LV systolic dysfunction, especially after uneventful 24 h-Holter monitoring.

## Supplementary Information

Below is the link to the electronic supplementary material.

Supplementary file1 (DOCX 3812 KB)

## References

[1] Sliwa K, Hilfiker-Kleiner D, Petrie MC, Mebazaa A, Pieske B, Buchmann E (2010). Current state of knowledge on aetiology, diagnosis, management, and therapy of peripartum cardiomyopathy: a position statement from the Heart Failure Association of the European Society of Cardiology Working Group on peripartum cardiomyopathy. Eur J Heart Fail.

[2] Bauersachs J, Konig T, van der Meer P, Petrie MC, Hilfiker-Kleiner D, Mbakwem A (2019). Pathophysiology, diagnosis and management of peripartum cardiomyopathy: a position statement from the Heart Failure Association of the European Society of Cardiology Study Group on peripartum cardiomyopathy. Eur J Heart Fail.

[3] Sliwa K, Petrie MC, van der Meer P, Mebazaa A, Hilfiker-Kleiner D, Jackson AM (2020). Clinical presentation, management, and 6-month outcomes in women with peripartum cardiomyopathy: an ESC EORP registry. Eur Heart J.

[4] Priori SG, Blomstrom-Lundqvist C, Mazzanti A, Blom N, Borggrefe M, Camm J (2015). 2015 ESC Guidelines for the management of patients with ventricular arrhythmias and the prevention of sudden cardiac death: The Task Force for the Management of Patients with Ventricular Arrhythmias and the Prevention of Sudden Cardiac Death of the European Society of Cardiology (ESC). Endorsed by: Association for European Paediatric and Congenital Cardiology (AEPC). Eur Heart J.

[5] Mallikethi-Reddy S, Akintoye E, Trehan N, Sharma S, Briasoulis A, Jagadeesh K (2017). Burden of arrhythmias in peripartum cardiomyopathy: analysis of 9841 hospitalizations. Int J Cardiol.

[6] Hoevelmann J, Viljoen CA, Manning K, Baard J, Hahnle L, Ntsekhe M (2019). The prognostic significance of the 12-lead ECG in peripartum cardiomyopathy. Int J Cardiol.

[7] Diao M, Diop IB, Kane A, Camara S, Kane A, Sarr M (2004). Electrocardiographic recording of long duration (Holter) of 24 hours during idiopathic cardiomyopathy of the peripartum. Arch Mal Coeur Vaiss.

[8] Duncker D, Haghikia A, Konig T, Hohmann S, Gutleben KJ, Westenfeld R (2014). Risk for ventricular fibrillation in peripartum cardiomyopathy with severely reduced left ventricular function-value of the wearable cardioverter/defibrillator. Eur J Heart Fail.

[9] Duncker D, Westenfeld R, Konrad T, Pfeffer T, Correia de Freitas CA, Pfister R (2017). Risk for life-threatening arrhythmia in newly diagnosed peripartum cardiomyopathy with low ejection fraction: a German multi-centre analysis. Clin Res Cardiol.

[10] Algra A, Tijssen JG, Roelandt JR, Pool J, Lubsen J (1991). QTc prolongation measured by standard 12-lead electrocardiography is an independent risk factor for sudden death due to cardiac arrest. Circulation.

[11] Sammani A, Kayvanpour E, Bosman LP, Sedaghat-Hamedani F, Proctor T, Gi WT (2020). Predicting sustained ventricular arrhythmias in dilated cardiomyopathy: a meta-analysis and systematic review. ESC Heart Fail.

[12] Can İ, Düzenli A, Altunkeser BB, Soylu A (2007). Peripartum cardiomyopathy presenting with complete heart block. Arch Turk Soc Cardiol.

[13] Yafi D, Noviani C, Saputri R, Purnawarman A, Andalas M, Yusmalinda Y (2021). Complete heart block in pregnancy: a case report. Indones J Cardiol.

[14] Luu M, Stevenson WG, Stevenson LW, Baron K, Walden J (1989). Diverse mechanisms of unexpected cardiac arrest in advanced heart failure. Circulation.

[15] Packer M (1992). Lack of relation between ventricular arrhythmias and sudden death in patients with chronic heart failure. Circulation.

[16] Faggiano P, d'Aloia A, Gualeni A, Gardini A, Giordano A (2001) Mechanisms and immediate outcome of in-hospital cardiac arrest in patients with advanced heart failure secondary to ischemic or idiopathic dilated cardiomyopathy. Am J Cardiol 87(5):655–7, A10–1 10.1016/s0002-9149(00)01450-8 11230859

[17] Kadish AH, Buxton AE, Kennedy HL, Knight BP, Mason JW, Schuger CD (2001). ACC/AHA clinical competence statement on electrocardiography and ambulatory electrocardiography. Circulation.

[18] Sana F, Isselbacher EM, Singh JP, Heist EK, Pathik B, Armoundas AA (2020). Wearable devices for ambulatory cardiac monitoring: JACC state-of-the-art review. J Am Coll Cardiol.

[19] Bisignani A, De Bonis S, Mancuso L, Ceravolo G, Bisignani G (2018). Implantable loop recorder in clinical practice. J Arrhythm.

[20] Hoevelmann J, Engel ME, Muller E, Hohlfeld A, Bohm M, Sliwa K (2022). A global perspective on the management and outcomes of peripartum cardiomyopathy: a systematic review and meta-analysis. Eur J Heart Fail.

[21] Duncker D, Veltmann C (2016) The wearable cardioverter/defibrillator–toy or tool? J Atr Fibrill 8(6) 10.4022/jafib.1367 PMC5089470 27909495

[22] Zhang S, Ching CK, Huang D, Liu YB, Rodriguez-Guerrero DA, Hussin A (2020). Utilization of implantable cardioverter-defibrillators for the prevention of sudden cardiac death in emerging countries: Improve SCA clinical trial. Heart Rhythm.

[23] Di Marco A, Brown PF, Bradley J, Nucifora G, Claver E, de Frutos F (2021). Improved risk stratification for ventricular arrhythmias and sudden death in patients with nonischemic dilated cardiomyopathy. J Am Coll Cardiol.

