# Supplementary material for: Effectiveness of implantable loop recorder and Holter electrocardiographic monitoring for the detection of arrhythmias in patients with peripartum cardiomyopathy
Source: Clin Res Cardiol. 2022 Sep 22;112(3):379–91. doi: 10.1007/s00392-022-02101-3 (PMC9998321; doi:10.1007/s00392-022-02101-3)
Supplement: Supplementary file 1 — Supplementary file1 (DOCX 3812 KB) [file 392_2022_2101_MOESM1_ESM.docx]

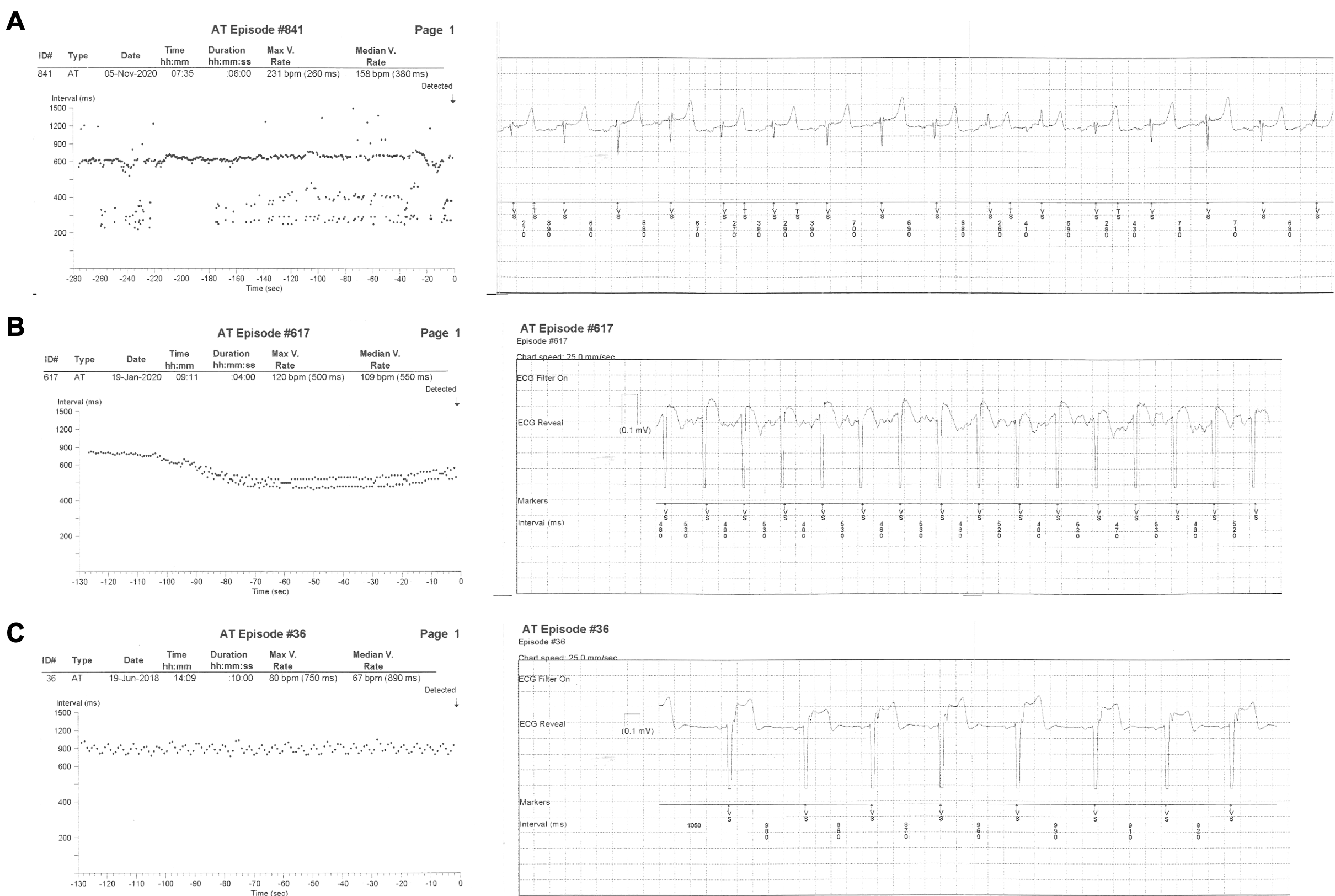

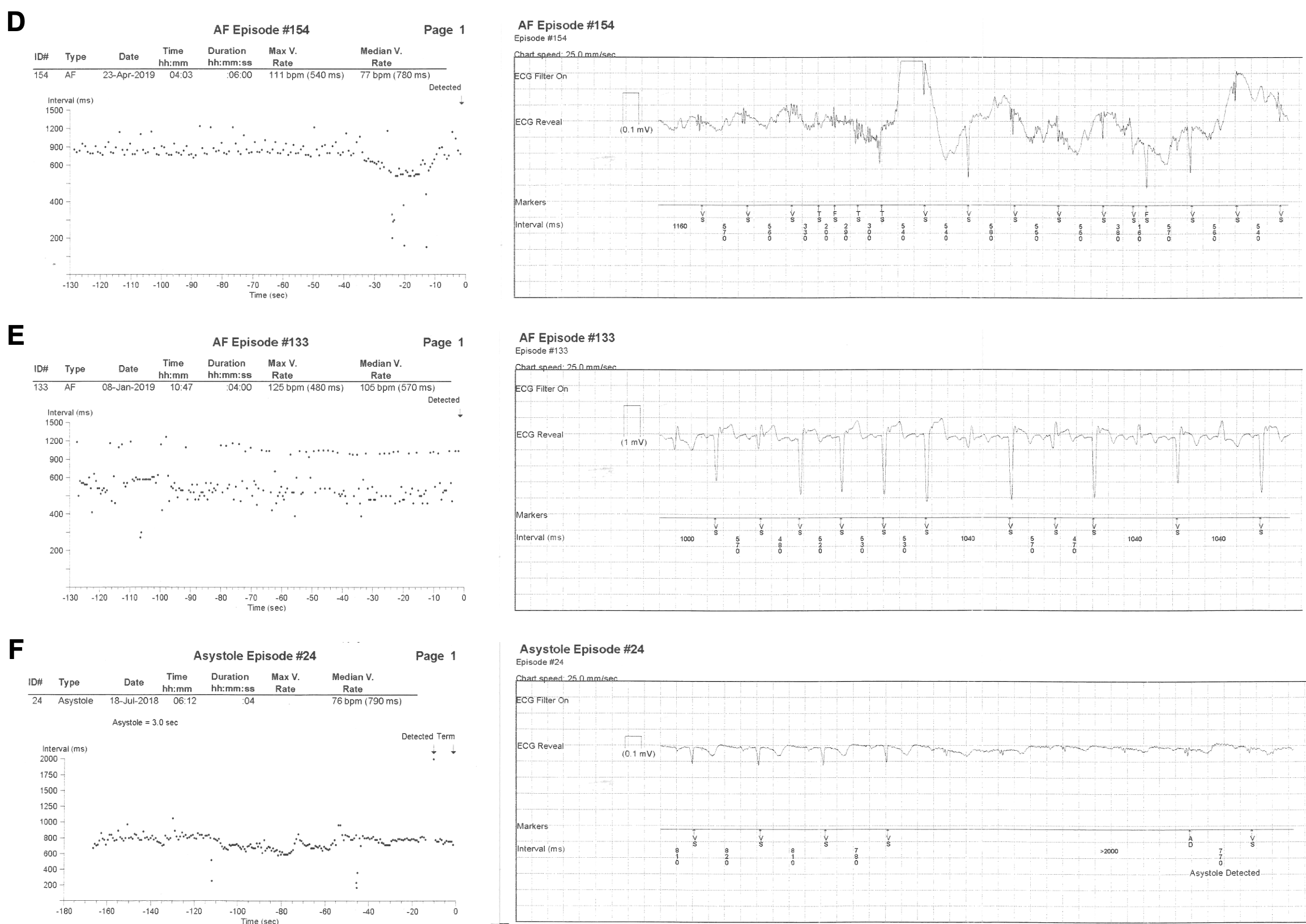


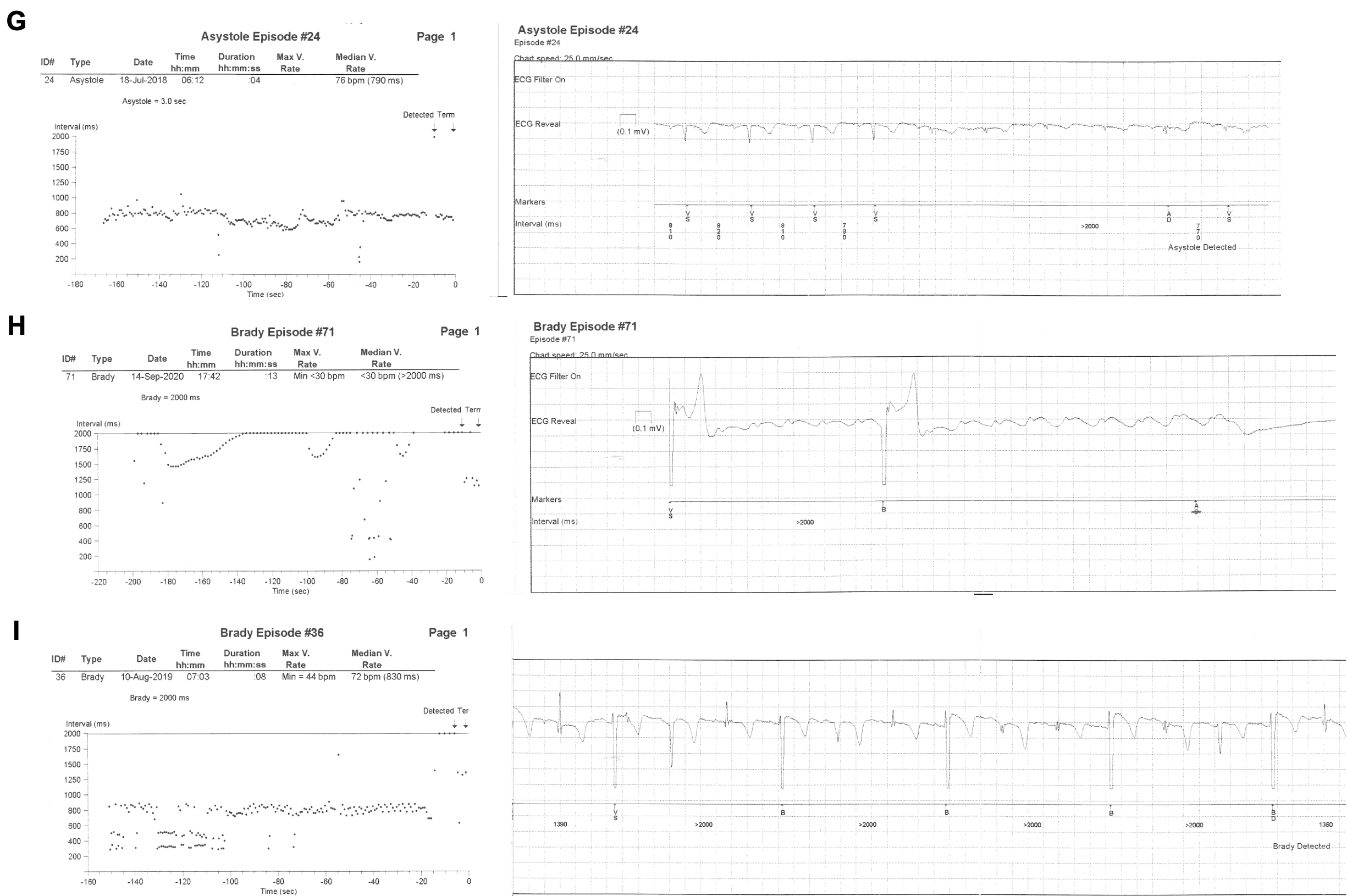


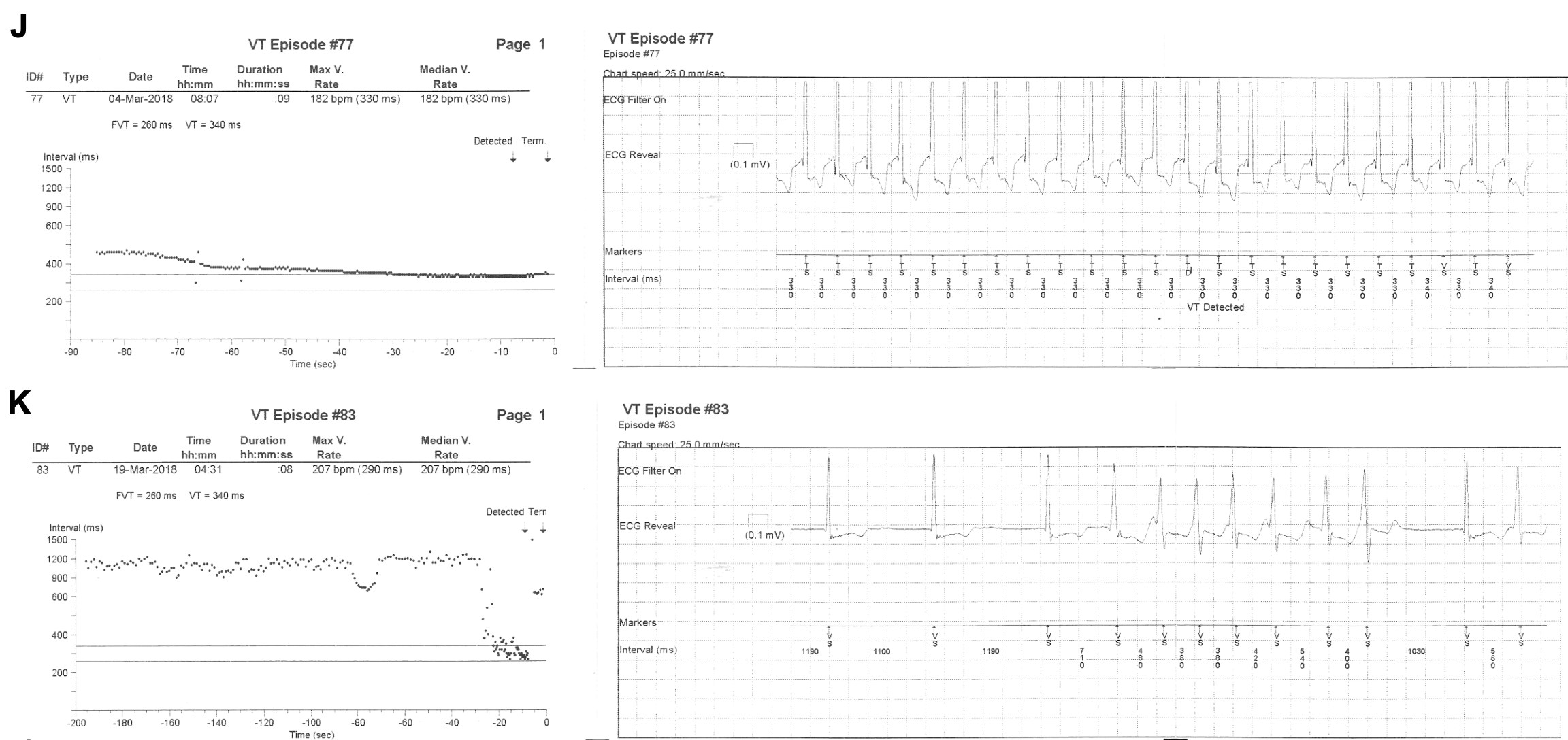


**Figure S1:** Adjudicated implantable loop recorder (ILR) interpretation: (A) Oversensing; (B) Sinus tachycardia; (C) T wave oversensing; (D) Artefact; (E) Undersensing; (F) Undersensing; (G) Undersensing; (E) Third degree AV block; (F) Undersensing; (J) Sinus tachycardia; (K) Non-sustained ventricular tachycardia.
